# High Prevalence and High Incidence of Coinfection with Hepatitis B, Hepatitis C, and Syphilis and Low Rate of Effective Vaccination against Hepatitis B in HIV-Positive Men Who Have Sex with Men with Known Date of HIV Seroconversion in Germany

**DOI:** 10.1371/journal.pone.0142515

**Published:** 2015-11-10

**Authors:** Klaus Jansen, Michael Thamm, Claus-Thomas Bock, Ramona Scheufele, Claudia Kücherer, Dieter Muenstermann, Hans-Jochen Hagedorn, Heiko Jessen, Stephan Dupke, Osamah Hamouda, Barbara Gunsenheimer-Bartmeyer, Karolin Meixenberger, HIV Seroconverter Study Group

**Affiliations:** 1 Robert Koch Institute, Berlin, Germany; 2 Labor Krone, Bad Salzuflen, Germany; 3 Medical Care Centre Jessen, Berlin, Germany; 4 Medical Care Centre Driesener Strasse, Berlin, Germany; National Institute of Child Health and Human Development, UNITED STATES

## Abstract

**Objectives:**

Men who have sex with men (MSM) are at higher risk for coinfection with hepatitis B virus (HBV), hepatitis C virus (HCV), and syphilis than the general population. HIV infection and these coinfections accelerate disease progression reciprocally. This study evaluated the prevalence and incidence of these coinfections in HIV1-positive MSM in Germany.

**Materials and Methods:**

As part of a nationwide, multicenter, prospective cohort study of HIV-infected MSM, plasma samples collected yearly were screened for HBsAg and antibodies to HBc, HBs, HCV, and syphilis. Samples with indications of active HBV or HCV infection were confirmed by polymerase chain reaction. Prevalence and incidence of each infection and incidence rates per study participant were calculated, and incidences over 4-year time intervals compared.

**Results:**

This study screened 5,445 samples from 1,843 MSM. Median age at HIV seroconversion was 33 years. Prevalences of active, cleared, and occult HBV, and of active/cleared HCV were 1.7%, 27.1%, 0.2%, and 8.2%, respectively, and 47.5% had been effectively vaccinated against HBV. Prevalence of antibodies to *Treponema pallidum* and of triple or quadruple sexually transmitted infections (STIs) were 39.6% and 18.9%, respectively. Prevalence of STI, cleared HBV, HBV vaccination, and history of syphilis differed significantly among age groups. Incidences of HBV, HCV, and syphilis were 2.51, 1.54, and 4.06 per 100 person-years, respectively. Incidences of HCV and syphilis increased over time. HCV incidence was significantly higher in MSM coinfected with syphilis and living in Berlin, and syphilis incidence was significantly higher for MSM living in Berlin.

**Discussion:**

Despite extensive HBV vaccination campaigns, fewer than 50% of screened MSM were effectively vaccinated, with a high proportion of HIV-positive MSM coinfected with HBV. High rates of STI coinfections in HIV-positive MSM and increasing incidences emphasize the need for better tailored campaigns for HBV vaccination and STI prevention.

## Introduction

Sexually transmitted infections (STIs) are a major health concern in patients infected with HIV. STIs can increase the risk of HIV infection [1–3] and worsen the course of disease [4]. Conversely, HIV infection can accelerate the course of concurrent STIs, including hepatitis B and hepatitis C, resulting in a faster progression to fibrosis and cirrhosis [5, 6] and making liver disease one of the most important non-AIDS causes of death of HIV-positive patients in the last few years [7–9]. Moreover, newly introduced direct-acting agents (DAAs) against hepatitis C virus (HCV) have shown severe drug–drug interactions with antiretroviral agents, complicating the treatment of both diseases [10, 11].

In Europe, men who have sex with men (MSM) represent an important proportion of persons living with HIV [12]. In Germany, MSM account for 66% of people currently living with HIV [13]. HIV-positive MSM are highly vulnerable to concurrent STIs [14–17], the rates of which have increased in Western countries since 2000 [18–20].

HIV-positive MSM are frequently coinfected with hepatitis B virus (HBV) and *Treponema pallidum* (TP), the agent causing syphilis [21–28]. European guidelines recommend screening sexually active HIV-positive MSM for HBV, HCV, and syphilis at diagnosis of HIV and at least annually thereafter [29]. In addition, German guidelines recommend that sexually active MSM and other immunocompromised persons be vaccinated for HBV [30]. However, because their immune responses are incomplete, HIV-positive individuals have lower rates of effective immunization against HBV [31–36]. In this population, also higher rates of occult HBV infections, producing only anti-HBc antibodies, were shown than the general population [37–40].

HCV coinfections of HIV-positive patients are observed mainly in HIV-positive individuals who inject drugs [41, 42]. Since 2000, outbreaks of acute HCV in HIV-positive MSM have been observed in large Western cities, including cities in Germany [43–49]. HCV can be transmitted sexually, such as through anal mucosal lesions caused by sexual practices such as fisting or through ulcerative STIs such as syphilis; through sharing paraphernalia for nasal and intravenous use of recreational drugs, such as heroin, cocaine, methamphetamines, and ketamine; or by proctosurgical interventions, e.g., in the treatment of condylomata [50–55].

The statutory reporting system for STIs in Germany includes only HIV and syphilis, as well as infections with HBV and HCV. As this reporting is anonymous on the national level, coinfections cannot be determined. Therefore, data on STI coinfections in MSM in Germany are based on sporadic studies, mainly in HIV-negative MSM [56–60]. In contrast, little is known regarding coinfections in HIV-positive MSM, although a few studies have assessed syphilis coinfection [25, 61, 62]. Furthermore, despite German recommendations that HIV-positive MSM be vaccinated against HBV, the success of this vaccination program has not been evaluated. In contrast, screening for HCV is recommended for sexually active HIV-positive MSM in Germany [29].

To provide further information about the prevalence and incidence of HBV, HCV, and syphilis coinfection among HIV-positive MSM in Germany, an extensive serological survey was performed. HIV-positive MSM with a known time of HIV seroconversion in Germany were assessed for coinfections with HBV, HCV, and syphilis as well as for effective immunization against HBV.

## Materials and Methods

### Study population, data, and sample collection

All analyses were based on a nationwide, multicenter, open, prospective cohort study of HIV 1-positive patients with a known or reliably estimated date of HIV 1 seroconversion. Patients were eligible for participation in the study if 1) having an acute HIV seroconversion (detectable HIV-1 RNA or p24 antigen combined with a negative or indeterminate ELISA result OR reactive HIV-1 ELISA combined with a negative or indeterminate immunoblot result with confirmation of complete seroconversion within six months) or 2) having a documented HIV seroconversion with at most a 3-year interval between the last negative and the first confirmed positive HIV antibody test. The blood sampling date of the first reactive test (acute seroconverters) or the arithmetic mean between the last negative and the first confirmed positive HIV antibody test (documented seroconverter) are considered as date of infection. The study period was from 15 June 1996 to 4 May 2012. Sociodemographic and clinical data, as well as blood samples from each participant, were collected at the time of enrollment and at yearly follow-ups. The methods of this study, which is assumed to be representative of MSM in Germany, except for those with an immigrant background, have been described in detail elsewhere [63–65]. The study protocol was initially approved in 2005 by the Ethics Committee of the Charité, University Medicine Berlin (EA2/105/05), with approval amended and confirmed in 2013. Participants provide their written informed consent to participate in this study. The ethics committee approved this consent procedure.

Patients were included if baseline sociodemographic data were collected and at least one plasma sample was available to estimate prevalence of coinfection. To estimate incidence, study participants had to be seronegative for a specific infection, with at least one additional plasma sample available.

### STI screening procedures

All EDTA-blood samples (10–20 mL) were sent by the treating physicians to the Robert Koch Institute (RKI). Buffy coat and plasma were separated by centrifugation, and plasma aliquots were stored at -70°C. Plasma samples were screened by the epidemiological laboratory of the RKI for HBsAg and for antibodies to HBc and HBs as markers of HBV infection, for anti-HCV antibodies as a marker for HCV infection, and for anti-*Treponema pallidum* antibodies as a marker for syphilis infection, using the Architect ci8200-system (Abbott, Wiesbaden, Germany). A cutoff of 0.5 was used to differentiate results of the TP-CLIA, as evaluated by Labor Krone (Bad Salzuflen, Germany), the consultant laboratory for Treponema [66]. Samples with signs of acute, chronic, or occult HBV infection were confirmed by qualitative polymerase chain reaction [PCR] (nested PCR using Hot Star Taq Master-Mix-Kit, Qiagen, Hilden, Germany). Samples testing positive for anti-HCV antibodies were tested by qualitative PCR (two-step PCR using the One Step RT-PCR-Kit and the Hot Star Taq Master-Mix-Kit, both Qiagen) and genotyped (Big Dye sequencing kits, Applied Biosystems, Darmstadt, Germany) by the RKI division for viral gastroenteritis and hepatitis pathogens and enteroviruses. Samples testing positive for anti-TP (Architect^®^ Syphilis TP, using a lower threshold of positivity of 0.5) were confirmed and their activity level evaluated by TPPA (Fujirebio, Tokyo, Japan), FTA-Abs IgM (Zeus, Raritan, USA), and RPR (Biokit, Barcelona, Spain) by Labor Krone. Samples were classified as positive for each specific infection as described in Table 1.

**Table 1:** Classification of samples being positive, by type of infection.

Type of infection	Serological results
Active Hepatitis B	anti-HBc (+), anti-HBs (−), HBs-AG (+), qual. HBV-PCR (+)
Cleared Hepatitis B	anti-HBc (+), anti-HBs (+), HBs-AG (−)
Occult Hepatitis B	anti-HBc (+), anti-HBs (−), HBs-AG (−), qual. HBV-PCR (+)
Effectively vaccinated against Hepatitis B	anti-HBc (−), anti-HBs (+,titer >10 mIU/mL), HBs-AG (−)
Active Hepatitis C	anti-HCV (+), qual. PCR (+)
Cleared Hepatitis C	anti-HCV (+), qual. PCR (−)
Active syphilis	TP-CLIA (+) AND TPPA ≥ 1:80 AND (FTA-Abs-IgM ≥ 1:40 OR RPR ≥ 1:8)
History of syphilis	TP-CLIA (+) AND TPPA ≥ 1:80 AND FTA-Abs-IgM ≤ 1:20 AND RPR ≤ 1:4

### Statistical analysis

The baseline sociodemographic and clinical characteristics of the study population were reported using descriptive statistics. Prevalences were calculated as the number of respective events divided by the number of study participants included in the study. Prevalences among age groups were compared using the Χ^2^-test or, if necessary, the Fisher’s exact test.

The dates of HBV, HCV, and syphilis seroconversion were defined as the date of drawing of the first sample testing positive for that infection. The total follow-up time per study participant, reported as person-years (PY) was calculated as the time between the date of HIV seroconversion and the date the last plasma sample was obtained. If the difference between the date of HIV seroconversion and the date of positivity for another infection was ≤ 14 days, that STI was considered present at the time of HIV seroconversion.

In analysing incidence, the follow-up time per study participant was defined as the time from the date of HIV seroconversion to the time of drawing the first sample positive for that infection or to the time of drawing the last sample testing negative for that infection. As reinfections are not statistically independent events, they were not considered in the analysis of incidence. Persons infected with HBV, HCV, or syphilis at the time of HIV seroconversion (first sample) were excluded from incidence analysis. The incidence of each infection was calculated as the number of infections divided by the cumulated number of PY during follow-up. Incidences, including 95% confidence intervals (CIs) were calculated for the complete study period, from 1996 to 2012, as well as for 4-year time intervals (1996–1999, 2000–2003, 2004–2007, and 2008–2012). Incidence rate ratios (IRR), including 95% CIs, were also calculated. All statistical analyses were performed using STATA (StataCorp LP, Collge Station, USA) version 13 software, with a p-value of <0.05 defined as statistically significant.

## Results

### Study population

The study screened 5,445 samples from 1,838 HIV-positive MSM, with the median number of samples per study participant for the overall study population being 2 (Table 2).

**Table 2:** Characteristics of the study population (for prevalence analysis), and of subpopulations assorted by coinfection with HBV, HCV, and syphilis (containing study participants available for incidence analyses).

Characteristic	Total population	HBV coinfection	HCV coinfection	Syphilis coinfection
Number of persons	1,838	468	1,784	1,280
Median age at HIV-seroconversion (range), yr	33 (17–76)	32 (14–68)	33 (17–76)	32 (17–68)
Median number of samples (range)	2 (1–12)	2 (1–11)	2 (1–12)	2 (1–12)
Total follow up-time (person years)	6,419	1,265	6,054	4,057
Median follow up-time (range), yr	2.6 (0–17.7)	1.7 (0–16.8)	2.6 (0–17.7)	2.5 (0–17.7)
Median time from HIV seroconversion to date of first sample obtained (range), yr	0.4 (0–14.0)	0.4 (0–12.8)	0.4 (0–14.0)	0.4 (0–13.9)
Median time from first to last blood sample (range), yr	1.9 (0–14.8)	1.0 (0–12.1)	1.9 (0–14.8)	1.9 (0–14.8)

The total number of PY was 6,419, with a median follow-up time per study participant of 2.6 years. The median time between the date of HIV seroconversion and the date of drawing the first blood sample was 143 days (0.4 years), and the median duration between drawing the first and last samples was 692 days (1.9 years).

Median age at HIV seroconversion was 33 years. Of the included MSM, 61.5% lived in Berlin, 11.2% in the federal states of North Rhine-Westphalia, 5.0% in Baden-Wurttemberg, and 4.6% in Bavaria.

### Prevalences

**Fig 1:**
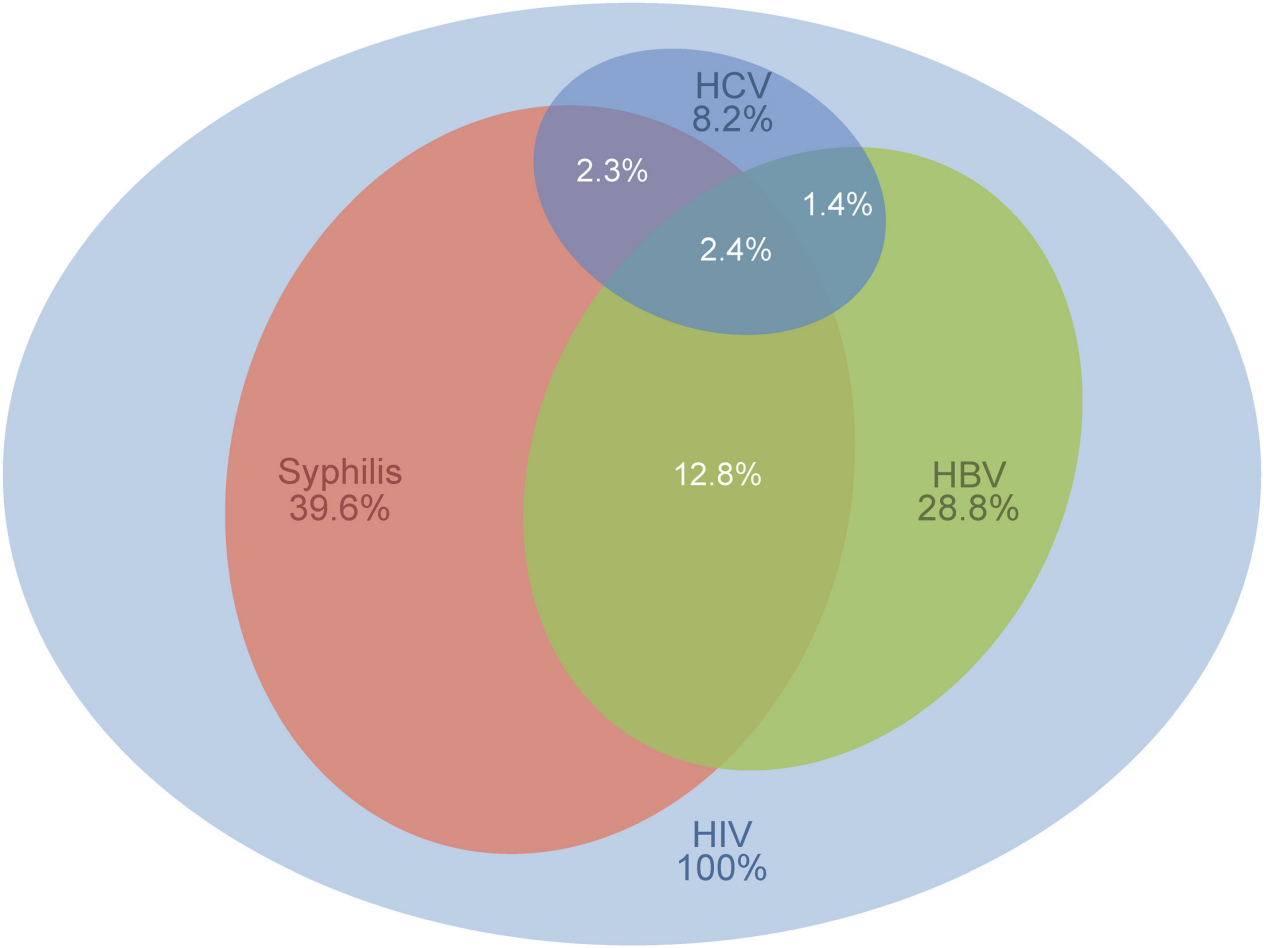
Prevalences of coinfections in MSM of the HIV seroconverter cohort. The figure shows coinfections with hepatitis B virus (HBV), hepatitis C virus (HCV), and syphilis. The areas of the ellipses correspond to the calculated proportions of the respective coinfections. White numbers: proportions of respective multiple infections. All percentages are relative to the total number of HIV-positive MSM (N = 1,838). The category “HBV” comprises HIV-positive MSM testing positive for an active, cleared, or occult HBV coinfection; the category “HCV” comprises HIV-positive MSM testing positive for an acute/chronic or cleared HCV-coinfection (for a definition on the basis of the serological testing results of these categories, see Table 1).

Of the HIV-positive MSM, 55.3% were coinfected with at least one of the other pathogens, HBV, HCV, or syphilis. Moreover, 16.6% were infected with two of these agents, and 2.4% were positive for all three (Fig 1).

At the time of blood drawing, 1.7% of the MSM cohort had active and 27.1% had cleared HBV infections, with 6.5% and 16.7%, respectively, diagnosed at the time of HIV seroconversion (Table 3).

**Table 3:** Prevalences of hepatitis B virus (HBV), hepatitis C virus (HCV), and syphilis in MSM with the HIV-seroconversion cohort, by infection status and age at HIV seroconversion (N = 1,838).

	Hepatitis B^1^					Hepatitis C^1^			Syphilis^1^			any STI^1^
	Negative	Active HBV	Cleared HBV	Occult HBV	Effectively vaccinated	Negative	Active HCV	Cleared HCV	Negative	Active Syphilis	History of Syphilis	
**All (n, %)**	432 (23.5%)	31 (1.7%)	498 (27.1%)	3 (0.2%)	874 (47.5%)	1,686 (91.8%)	74 (4.0%)	78 (4.2%)	1,109 (60.4%)	231 (12.5%)	498 (27.1%)	1,020 (55.3%)
**Age (%)**												
p- value^2^	0.160	0.458	<0.001	0.229	<0.001	0.429	0.486	0.130	0.007	0.872	0.017	<0.001
< 25 years	23.9%	0.4%	12.6%	0%	62.3%	91.9%	5.7%	2.4%	69.2%	11.3%	19.4%	39.3%
25–34 years	25.8%	1.9%	21.7%	0%	50.3%	93.0%	3.5%	3.5%	61.6%	12.2%	26.2%	52.5%
35–44 years	20.7%	2.1%	33.6%	0.5%	43.1%	90.2%	3.9%	5.8%	56.1%	13.7%	30.2%	62.3%
45–54 years	20.1%	1.3%	46.5%	0%	32.1%	91.2%	3.8%	5.0%	56.6%	12.0%	31.4%	68.0%
≥ 55 years	29.0%	0%	48.4%	0%	22.6%	90.3%	6.5%	3.2%	58.1%	12.9%	29.0%	64.5%

^1^For classification of samples as positive for each specific infection see Table 1

^2^Comparison by X^2^-test or Fisher’s exact test, where necessary.

Of the 47.5% of MSM effectively vaccinated against HBV, 25.4% had been vaccinated prior to HIV seroconversion, and 28.6% within the first 6 months after HIV seroconversion. Among the latter study participants, the median duration between HIV seroconversion and the date of drawing of the first sample showing effective HBV-vaccination was 9 months (range 1–179 months).

Of the HIV-positive MSM, 2.5% (46) were positive only for anti-HBc, suggesting occult HBV infection. Three of these study participants were HBV DNA positive, confirming occult HBV infection, all 6–18 months after HIV seroconversion. All three were negative for the anti-HCV antibody. Follow-up samples in six other study participants were positive for anti-HBc, but occult HBV infection could not be confirmed by HBV PCR. However, samples previously drawn from these six study participants tested positive for anti-HBc and anti-HBs concurrently, suggesting cleared HBV infection, and the follow-up samples tested solely positive for anti-HBc. The anti-HBs titers of all of these samples were very close to the cut-off of the Architect anti-HBs assay. These samples were regarded as showing cleared HBV infection. The prevalence of cleared HBV infection and of effective vaccination differed significantly among age groups (Table 3).

Overall, 8.2% of MSM were positive for HCV infection. Of these, 48.7% had a viremic sample after HIV seroconversion. HCV genotype 1 was most frequent (71.6%), followed by genotypes 4 (19.2%), 3 (6.8%), and 2 (2.7%). Of the HIV-positive MSM testing positive for HCV, 10.5% were coinfected with HCV at the time point of HIV seroconversion. Among study participants first positive for anti-HCV after HIV seroconversion, the median time from HIV seroconversion to the first sample taken positive for anti-HCV antibody was 30 months (range 1–190 months).

Of the HIV-positive MSM, 39.6% were positive for syphilis coinfection, including 31.6% with active syphilis after HIV seroconversion. Of the syphilis-positive MSM, 10.7% were already coinfected at the time of HIV seroconversion. Among MSM first positive for anti-TP antibodies after HIV seroconversion, the median time between HIV seroconversion and the date of drawing the first blood sample positive for anti-TP antibody was 11 months (range 1–194 months). The prevalence of a history of syphilis differed significantly among age groups (Table 3).

The prevalence of having any STI differed significantly among age groups (Table 3) and was significantly higher among study participants living in Berlin than in other cities (58.4% vs. 50.4%; p-value = 0.001).

### Incidences

The total study population was followed-up for 6,419 PY, with follow-up times differing among specific infections, owing to a different number of MSM being at risk and therefore available for incidence analysis (Table 1). During the complete study period from 1996 to 2012, the incidences of HBV, HCV, and syphilis among persons at risk for each disease were 2.51, 1.54, and 4.06 per 100 PY, respectively.

**Fig 2:**
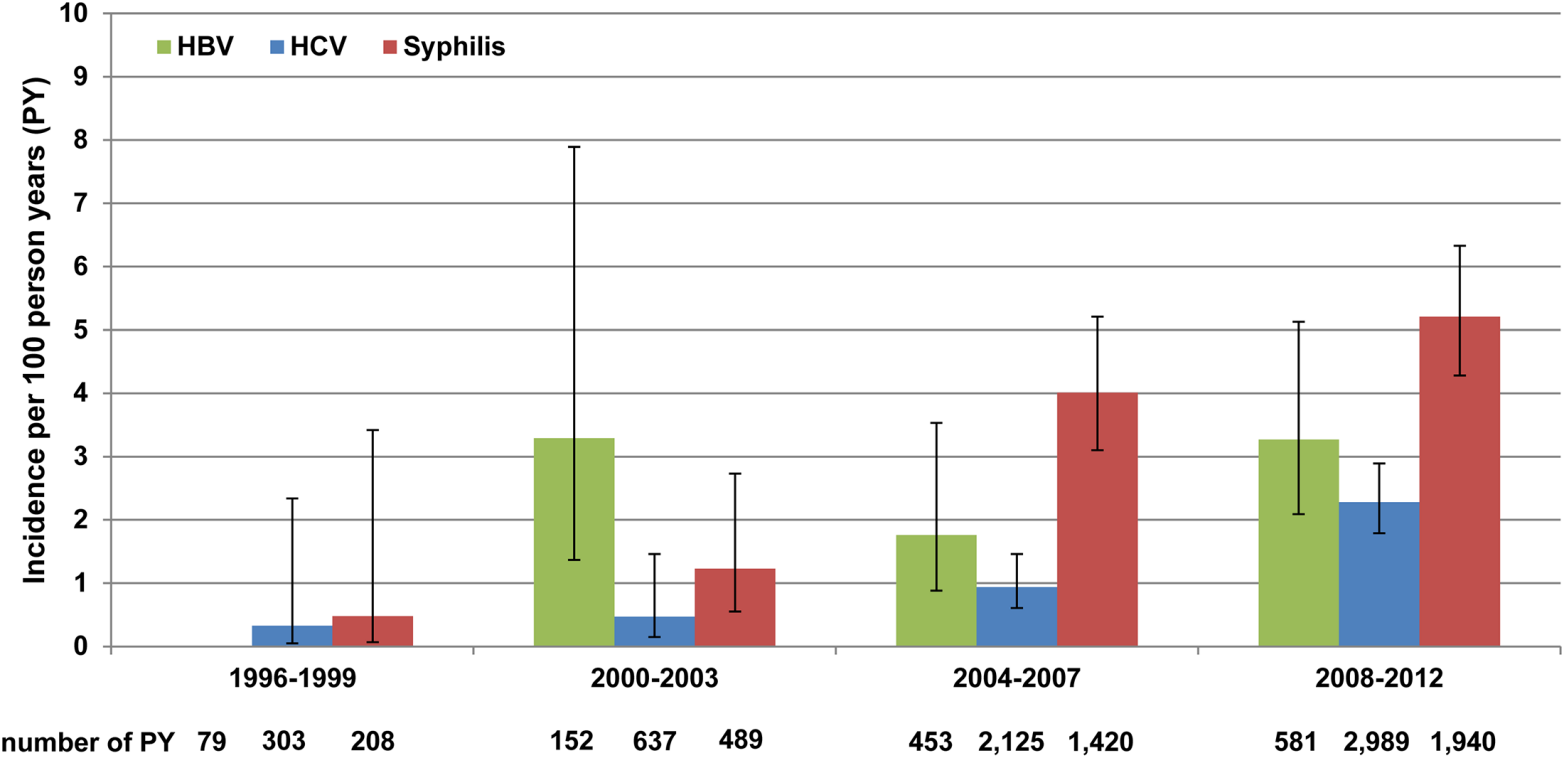
Incidences of coinfections in MSM of the HIV seroconverter cohort, by time period. Incidences of hepatitis B virus (HBV), hepatitis C virus (HCV), and syphilis infection were calculated per 100 person-years (PY). Whisker graphs show lower and upper limits of calculated 95% confidence intervals. Time under follow-up (number of PY) per infection and time period are indicated below the figure.

When the study period was divided into 4-year time intervals, the incidence of HBV fluctuated between 0 and 3.29/100 PY (Fig 2).

The incidence of HCV increased over time, from 0.33/100 PY in 1996–1999 to 2.28/100 PY in 2008–2012. Similarly, the incidence of syphilis increased over time, from 0.48/100 PY in 1996–1999 to 5.21/100 PY in 2008–2012, with 95% CIs overlapping. The greatest increases in the incidences of HCV and syphilis occurred from 2004–2007 to 2008–2012.

**Fig 3:**
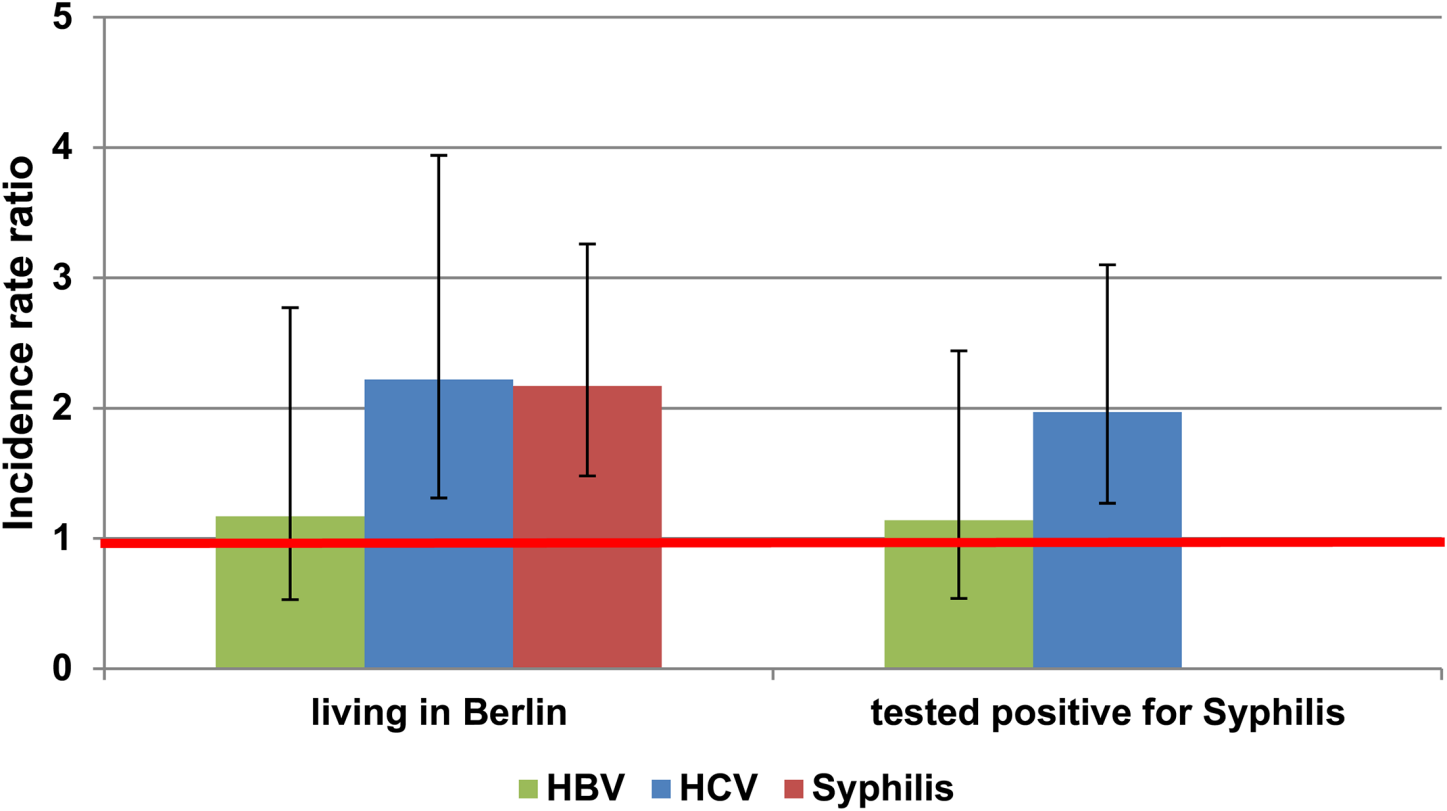
Incidence rate ratios (IRR) of coinfections in MSM of the HIV seroconverter cohort. IRR for infection with hepatitis B virus (HBV), hepatitis C virus (HCV), and syphilis were calculated for persons living in Berlin and those living elsewhere in Germany and in persons testing positive for syphilis at any time point or and those always testing negative. Whisker graphs show lower and upper limits of calculated 95% confidence intervals.

The incidence of HBV was slightly, but not significantly higher, among MSM living in Berlin than in those living elsewhere in Germany (IRR = 1.17; p = 0.35; Fig 3) and than among those who did not test positive for syphilis during the study period (IRR = 1.14; p = 0.35).

The incidence of HCV was two-fold higher among MSM living in Berlin than elsewhere in Germany (IRR = 2.22, p<0.01) and among those who did than did not test positive for syphilis during the study period (IRR = 1.97, p<0.01). The incidence of syphilis was about two-fold higher among MSM living in Berlin than elsewhere in Germany (IRR = 2.17, p<0.01).

## Discussion

This study was the first extensive screening in Germany of plasma samples from HIV 1-positive MSM with known date of HIV 1-seroconversion for coinfection with HBV, HCV, and syphilis. The prevalences of HBV (28.8%), HCV (8.2%), and syphilis (39.6%) coinfections were high, as was the prevalence of infections with two or three of these STI (18.9%). The incidences of HBV and HCV were about 50–100% higher in MSM positive than negative for syphilis. Furthermore, the incidences of HCV and syphilis were significantly higher among MSM living in Berlin than among those living elsewhere in Germany.

### Hepatitis B

The proportion of HIV-positive MSM with an active acute or chronic HBV infection was low, only 1.7%; these individuals, however, were able to transmit HBV to other individuals [16, 23]. In contrast, a much higher proportion of study participants had been previously infected with HBV, most after becoming HIV positive. The incidence of HBV showed no trend over time, did not differ between study participants living in Berlin and those living elsewhere in Germany, and was similar in study participants who were and were not coinfected with syphilis. Thus, coinfection with HBV differed clearly from coinfections with HCV and syphilis in this study population. The reasons for these fluctuations remain unclear, especially because the prevalence of HBV among HIV-infected MSM in Germany did not change substantially over time and there were no important changes in recommendations for HBV vaccination, diagnostic and treatment.

More than half of the HIV-positive MSM population had not been effectively vaccinated against HBV, with a quarter susceptible to infection. This proportion of effectively vaccinated MSM was particularly low in older age groups, who had not been vaccinated during childhood or adolescence. This finding was in good agreement with the results of a large internet survey of self-reported HBV infection and vaccination status among MSM in Europe [67]. As HBV vaccination campaigns for MSM and for HIV-positive patients are long-standing in Germany [30], our results suggest that this public health task has not been effectively implemented. Increases in liver-related morbidity and mortality rates among HIV-infected patients coinfected with HBV [68, 69] suggest the importance of effective immunization against HBV among these individuals. Ideally, individuals should be vaccinated for HBV prior to be at risk for becoming HIV positive because the effectiveness of vaccination is poorer in HIV-positive than HIV-negative individuals [31–36]. If not previously vaccinated, HIV-positive individuals should be immunized against HBV while having a low HIV viral load, as HIV viremia shortens the duration individuals remain anti-HBs positive [70].

As anti-HBs antibodies can be lost several years after effective HBV vaccination of HIV-positive patients [34], we cannot state with certainty that study participants negative for anti-HBs had not been previously vaccinated. Regardless, these study participants were susceptible to HBV, suggesting the need for strategies that boost immune responses in immunocompromised individuals [32, 34, 35, 71].

Occult HBV infections, confirmed by PCR, were rare in our cohort (0.2%). Of all study participants positive only for anti-HBc, 6.5% were confirmed by PCR, with the prevalence of occult HBV in our study being lower than in most other reports [40, 72, 73]. Despite the substantial proportion of MSM infected with HCV, none of the study participants with occult HBV infection was positive for anti-HCV. Thus, in contrast to previous findings [40], our results provided no evidence for an increase in occult HBV infection in HCV-coinfected study participants, although the sample sizes of the respective subpopulations were small. Infection status was changed in six study participants, from cleared to occult HBV infection in consecutive sample, owing to reductions in their anti-HBs titers to just below the cut-off of the Architect anti-HBs assay. These study participants may also have cleared HBV infections, with anti-HBs titers reduced over time because of their immune deficiency. In contrast, these individuals may have had actual occult infections with very low HBV-DNA at the time the samples were taken. This ambiguity indicates the need to investigate these titers in HIV-positive patients with occult HBV infection and to partly adjust cut-off levels individually to obtain a valid HBV diagnosis.

### Hepatitis C

The prevalence of HCV (active and cleared) in HIV-positive MSM in our study 27-fold higher than in the general German population [74] and nearly equal to the prevalence of 8.8% self-reported by HIV-positive MSM in a large German internet survey [60]. Other European studies in HIV-positive MSM found both lower [16, 75–77] and higher [46, 47] HCV prevalences, ranging from 4.2% to 17.8%. Studies reporting higher incidences combined antibody testing and HCV-specific PCR, thus likely included more study participants with early-stage infection and delayed antibody responses.

The incidence of HCV infection in our study population was within the range reported in other studies [75, 77–80]. The outbreaks of HCV infection in HIV-positive MSM in Western cities since 2000 [43–49] likely also affected our study population, as the incidence of HCV was seven times higher in 2008–2012 than in 1996–1999. Interestingly, the strongest absolute increase in HCV-incidence in our study population occurred between 2004–2007 and 2008–2012, later than the first reported outbreaks of HCV in HIV-positive MSM in large Western cities. Nevertheless, HCV incidence increased over the entire study period.

Most HCV-positive participants were coinfected with HCV after HIV seroconversion. As we did not have any data on risk behaviour, we could not analyze specific risk factors for HCV coinfection. However, two thirds of these HCV-coinfected MSM were also infected with HBV and/or syphilis, indicating sexual transmission of HCV. Furthermore, we found that the incidence of HCV coinfection was more than two-fold higher among MSM living in Berlin than elsewhere in Germany. Berlin is a current hotspot of MSM sex tourism in Europe, HCV transmission networks in Berlin were reported [43, 44, 48]. This finding is also applicable when comparing HCV incidences between HIV-positive MSM with or without syphilis coinfection. Hence, physicians treating sexually active HIV-positive MSM living in or traveling to Berlin should counsel these patients about the risks of HCV and test them regularly for HCV infection.

The high prevalence of HCV-coinfected MSM in our study supports the need for yearly HCV screening of HIV-positive, sexually active MSM, as described in recent guidelines [29]. This knowledge may help improve counseling and optimize HCV treatment by earlier initiation during the acute phase of the infection. This, in turn, may reduce viral load among this population and decrease further transmission of this virus. A 12-month screening program for HCV antibodies among HIV-positive MSM in the US, in combination with liver function tests, was recently shown to be cost effective in the US [81].

About half of the HCV-coinfected study participants were viremic (PCR positive) after HIV seroconversion. These patients may benefit from concurrent treatment for HCV along with antiretroviral therapy against HIV. As HCV-genotypes 1 and 4 were predominant, making standard therapy with interferon and ribavirin less effective, treatment with newly introduced DAAs may result in the successful elimination of HCV from these patients. These interferon-free regimens may be especially effective in HIV-positive patients, as these patients show a faster progression to fibrosis and cirrhosis than HIV-negative patients [5, 6] and DAAs have high virological efficacy in HIV-positive patients [82] and good tolerability. Because of their current high costs, treatments with these agents are limited, including in Western countries.

### Syphilis

The prevalence of syphilis was the highest of the three STIs screened. These findings are in good agreement with other reports on syphilis epidemics in both MSM [19, 26, 59, 67, 83] and HIV-positive MSM [15–17, 25, 84]. In 2013, 81.4% of syphilis-infected patients in Germany were found to be MSM, based on the statutory reporting system [85]. More detailed analyses of statutory data showed some evidence for an overlap between both infections in MSM in Germany, findings likely owing to their sexual risk behavior [25]. In our study, 89.3% of MSM coinfected with syphilis became infected after the time of HIV seroconversion, suggesting that this type of risk behavior continues after subjects become HIV positive. This finding emphasizes the importance of counseling on STI risks within the context of HIV treatment. As HIV-positive MSM in Germany are usually in close contact with physicians who specialized in HIV treatment, these physicians may play an important role in STI prevention.

Interestingly, and in contrast to previous findings [28], the overall prevalence of antibodies to *Treponema pallidum*, as well as the prevalence of active syphilis, were higher in older age groups. Our results indicated that MSM between ages 35 and 55 years were at higher risk of having a history of syphilis than other age groups, but not for active syphilis. As residual antibodies for syphilis can remain for a long time, especially in HIV-positive patients, our data gave no evidence for a higher risk of infection with syphilis in older age groups, but pictures an accumulation of residual antibodies over the life course of MSM.

The overall incidence of syphilis, 4.06/100PY, was also in line with previous data [22]. Similar to HCV, the incidence of syphilis increased over time and was two-fold higher in MSM living in Berlin than elsewhere in Germany. This finding is in good agreement with statutory data, showing that the incidence of syphilis among MSM in Germany has been increasing since 2010 [85].

More than half of HIV-positive MSM coinfected with HCV were also coinfected with syphilis, suggesting that each can act as a reciprocal marker for the other. Thus, the occurrence of either HCV or syphilis in HIV-positive MSM should provoke screening for the other infectious agent.

The study data strongly support guidelines recommending that HIV-positive MSM be screened for syphilis, especially because studies in HIV-positive MSM showed that about 50% of syphilis-coinfected individuals were asymptomatic for syphilis [22, 28, 86].

### Strengths and Weaknesses

This study is the first large serological survey of HIV-positive MSM in Germany to assess coinfections with HBV, HCV, and syphilis. A special strength is that serum samples were not collected in relation to assessments of specific sexual risk including STI screening, but during routine medical care for HIV infection. Because of the cohort structure, another benefit of this study was the ability to relate the date of STI diagnosis with the known date of HIV seroconversion.

The study also had several weaknesses. First, the prevalence and incidence of HCV infection may have been underestimated because of a delayed antibody response to acute infection, especially when subsequent blood samples were not available [87–89], or to a loss of antibodies between two consecutive blood drawings. Conversely, HCV infection may have been overestimated, as samples positive for anti-HCV were not confirmed by Western blotting, owing to the epidemiological focus of this study. Thus, although some positive anti-HCV test results may have been false positives, the high specificity (99.6%) of the testing system used [90] suggests that overestimation is unlikely.

Following the literature, there is a potential for occult HCV [91–93], but this was not looked for in this study.

The date of HIV seroconversion was estimated for a larger proportion of study participants as the mean between a documented last negative and a first positive HIV test with at most a 3-year interval in between. This could cause some inaccuracies regarding analyses that include time of HIV seroconversion.

As the study is laboratory orientated, no data on behavior, clinical characteristics, or treatment of HBV, HCV, and syphilis, were collected. Thus, we could not calculate a risk factor model by which the study population acquired an STI. Therefore, in 2014, we introduced a new and extensive data module on HBV and HCV infection and their treatment into the cohort study.

### Recommendations

The high prevalence of HBV, HCV, and syphilis in our population of HIV-positive MSM underscores the strong need for ongoing and comprehensive STI prevention among HIV-positive MSM in Germany. This population should be screened regularly for all three indications, as recommended in current guidelines. Ongoing efforts to minimize the proportion of HIV-positive MSM not being vaccinated effectively for HBV and remaining susceptible to HBV infection are of great importance, especially because HIV-positive patients are at risk for severe liver damage.

MSM, or at least those sexually active or diagnosed with another STI diagnosis, should be screened for HCV infection. An analysis in the US showed that screening for HCV, in combination with liver function tests, was cost effective [81]. Although a similar analysis has not been performed in Germany, the similarity of parameters suggests that this type of screening program may be equally cost effective. HCV-related phylogenetic analyses may help better understand its epidemiology in HIV-infected individuals and shape more effective preventative approaches. Finally, from a public health perspective, broad use of newly developed DAAs to treat HCV may reduce HCV viral load in this population and reduce the risks of HCV transmission.

The medical care of HIV-positive MSM in Germany suggests that private practitioners as well as outpatient clinics specialized in medical care for HIV may be important in enhancing vaccination coverage against HBV as well as optimal screening for STI of HIV-positive MSM. Our results suggest that special emphasis be given to MSM living in or traveling to Berlin.
